# Rationally designed chromosome fusion does not prevent rapid growth of *Vibrio natriegens*

**DOI:** 10.1038/s42003-024-06234-1

**Published:** 2024-05-02

**Authors:** Lea Ramming, Daniel Stukenberg, María del Carmen Sánchez Olmos, Timo Glatter, Anke Becker, Daniel Schindler

**Affiliations:** 1 Max Planck Institute for Terrestrial Microbiology, Marburg, Germany; 2 Department of Biology, Philipps-Universität Marburg, Marburg, Germany; 3 Center for Synthetic Microbiology (SYNMIKRO), Philipps-Universität Marburg, Marburg, Germany

**Keywords:** Microbial genetics, DNA replication

## Abstract

DNA replication is essential for the proliferation of all cells. Bacterial chromosomes are replicated bidirectionally from a single origin of replication, with replication proceeding at about 1000 bp per second. For the model organism, *Escherichia coli*, this translates into a replication time of about 40 min for its 4.6 Mb chromosome. Nevertheless, *E. coli* can propagate by overlapping replication cycles with a maximum short doubling time of 20 min. The fastest growing bacterium known, *Vibrio natriegens*, is able to replicate with a generation time of less than 10 min. It has a bipartite genome with chromosome sizes of 3.2 and 1.9 Mb. Is simultaneous replication from two origins a prerequisite for its rapid growth? We fused the two chromosomes of *V. natriegens* to create a strain carrying one chromosome with a single origin of replication. Compared to the parental, this strain showed no significant deviation in growth rate. This suggests that the split genome is not a prerequisite for rapid growth.

## Abstract (web summary)

The fastest growing bacterium known is *Vibrio natriegens*. We have fused the two chromosomes of *V. natriegens* to create a single chromosome strain. Our results show that the bipartite genome is not a prerequisite for its rapid growth

## Introduction

Every cell must replicate its genome prior to cell division. Canonical initiation of DNA replication in bacteria is primed at a single origin of replication (*ori*) from which the chromosome (chr) is replicated bidirectionally once per cell cycle^1^. The processive rate of DNA polymerase is approximately 1000 bp per second^2^. Since DNA must be replicated prior to completing the cell cycle, replication rate can determine generation time. To overcome this bottleneck, some bacteria have evolved a system of overlapping replication cycles to increase growth rates^3^. Notably, initiation of DNA replication still takes place only once per cell cycle but daughter cells are already born with replicating chromosomes^4,5^. By maintaining high *ori:ter* ratios, *Escherichia coli*, achieves doubling times of 20 min^3^. The genome of *E. coli* is organized in a single chromosome with a size of 4.6 Mb. In contrast, the human pathogen *V. cholerae* has a bipartite genome with chromosome sizes of 3.0 Mb and 1.1 Mb, respectively^6^. *V. cholerae* was reported to achieve doubling times faster than *E. coli*, and the bipartite genome may be a reason for its faster growth^7^. The *ori1* of the larger chr1 is highly similar to the *oriC* of *E. coli*. The *ori2* of chr2, however, has a different architecture and presumably a plasmid-based origin with its own partitioning system (ParAB_2_) and initiator protein (RctB)^8^. The DNA replication of the two chromosomes is coordinated within the cell cycle leading to an evolutionary conserved termination synchrony in *Vibrionaceae*^9^. The orchestration is achieved by the chr2 replication triggering site (*crtS*) located on chr1^10,11^. Once *crtS* is replicated, replication of chr2 is initiated, passively coordinating termination synchrony. In an earlier study, researchers were able to engineer the *V. cholerae* genome into a single chromosome strain and a strain with equal-sized chromosomes, the strain with a single chromosome was termed MCH1 (MonoCHromosomal *V. cholerae*)^12^. Interestingly, both strains exhibit an increased doubling time in defined rich media of 26 and 34.8%, respectively. This increase in doubling time is thought to be due to challenges with cell division licensing in MCH1, presumably due to the nucleoid occlusion system caused by misplacement of the SlmA protein^13,14^ and not just, as one might think, because of the increased size of the replicon.

In recent years, *Vibrio natriegens* has received increased attention because of its rapid growth with reported doubling times <10 min, despite already being known for >60 years^15–17^. *Vibrio natriegens* has a bipartite genome with chromosome sizes of 3.2 Mb and 1.9 Mb, respectively. Researchers have identified a set of 587 *V. natriegens* genes required for rapid growth in rich media, identified by CRISPRi screening^18^. Among those are genes encoding ribosomal proteins, metabolic genes, and genes encoding for DNA polymerase. As expected, the reduction of essential proteins such as the DNA polymerase results in reduced growth rate. However, this result does not determine whether DNA replication is a rate-limiting factor for *V. natriegens’* rapid growth.

To investigate the role of DNA replication on maximum cellular growth rate, we reconfigured the chromosomal architecture to require all replication to fire from a single origin. We created and characterized the *V. natriegens* strain synSC1.0 (synthetic single chromosome v.1.0), a strain derivative of ATCC14048 with its two chromosomes fused into a singular chromosome. We prove by replication pattern analysis that the replication of the fused chromosome is initiated from a single origin of replication. We were expecting increased doubling times in synSC1.0 based on the existing reports of work with *V. cholerae* MCH1. However, our results indicate that the consequences of extended DNA replicon length in synSC1.0 are negligible for its rapid growth. The strain synSC1.0 will allow novel approaches to study chromosome biology in this rapidly growing bacterium. *V. natriegens* may be a suitable alternative to *V. cholerae*, the currently most well-studied model organism for bipartite microbial genomes, allowing to study chromosome biology without the risk of infections. Besides its application in basic research synSC1.0 may be an interesting chassis for synthetic biology and applied research, e.g. for hosting an additional synthetic chromosome based on the *ori2* region of the native chr2.

## Results

### Construction and validation of a single chromosome *V. natriegens* strain

**Fig. 1:**
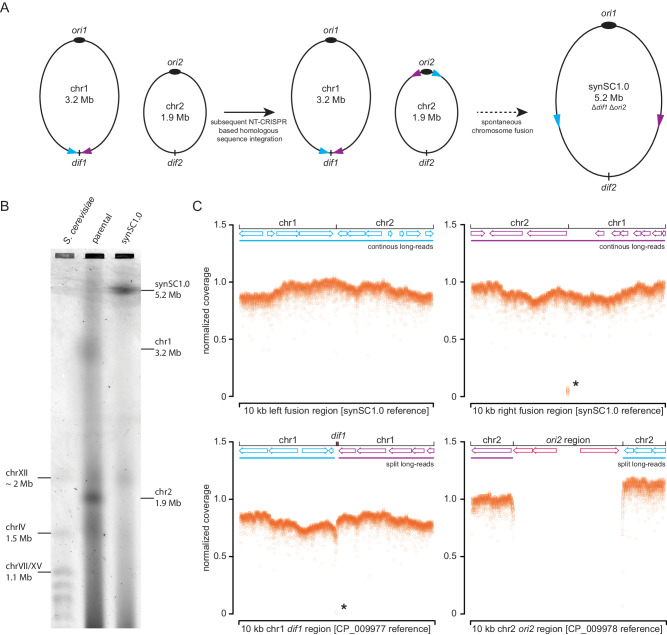
Construction and validation of the *V. natriegens* strain synSC1.0. **A** Scheme of the developed strategy for chromosome fusion and large-scale genome engineering of *V. natriegens*. NT-CRISPR is utilized to subsequently integrate homologous flanking sequences into the second chromosome in the initial step. The picked homologous sequences and their orientation are indicated by blue and purple arrows. We planned to select for the fused chromosome with deleted *dif1* and *ori2* with double-strand breaks from CRISPR/Cas9. To our surprise, the chromosome fusion already occurred in the initial step while integrating the second homologous region. Sizes are not to scale and differences in chromosome sizes are due to truncation. **B** PFGE shows two bands for the parental strain with sizes of approx. 3.2 and 1.9 Mb representing chromosome 1 and 2, respectively. For synSC1.0, only a single band with an increased size of approx. 5.2 Mb is visible. The sizes were estimated by using *S. cerevisiae* as reference. **C** Long-read sequencing of synSC1.0 confirms fusion of the two chromosomes and deletion of *dif1* and the *ori2* region. The two top panels show the confirmation of the left and right fusion sites visualized by the normalized coverage for a 10 kb window using the designed synSC1.0 reference. The right panel indicates the small deletion of 38 nucleotides indicated by an asterisk. The two bottom panels show the data plotted against the CP_009977 and CP_009978 references respectively to validate the deletion of *dif1* (left panel, indicated by an asterisk) and the *ori2* region (right panel). X-axis resembles a 10 kb window. Zero values are not plotted. The top of each graph contains a scheme open reading frame annotations of the genetic content in this region, while the lines indicate long-reads either spanning the whole region (continuous color, top panel) or continue at different coordinates (split color, lower panel) based on the indicated fusion sites and reference sequence. An enlarged figure with annotations of the genetic content is provided in the Supplementary Information (Fig. S1). Blue and purple indicate the left and right fusion regions, respectively, and deleted regions are annotated in red.

Assuming a replication speed of 1000 bp/s, the replication time for the *E. coli* genome is approx. 40 min, which is in line with the literature^2^. Transferring this replication speed to the bipartite genome of *V. natriegens* would result in a replication time of 27 min for the larger chr1 (3.2 Mb), while in a strain with fused chromosomes (5.2 Mb) the replication time would be approx. 43 min, an increase of around 60%. To test if DNA replication is the rate-limiting factor of *V. natriegens* rapid growth, we fused the two chromosomes by replacing the deletion-induced filamentation (*dif*) site^19^ of chr1 with the whole chr2 except for the *ori2* region. *dif* is important for chromosome dimer resolution via the site-directed recombinases XerC/D^20,21^. The fused chromosome possesses the origin of replication of the first chromosome (*ori1*) for initiation of DNA replication and the *dif* site of the second chromosome (*dif2*) for chromosome dimer resolution (*cf*. Fig. 1A)^22^. The *ori2* region contains the genes for the partitioning system ParAB_2_ and the chr2 replication initiator protein RctB. The strain construction was performed utilizing our earlier published NT-CRISPR procedure (Fig. 1A). In two subsequent editing steps, we integrated homologous sequences of chr1 flanking the *dif1* site upstream and downstream of the *ori2* region in chr2. Initially we planned to enforce chromosome fusion through a gRNA directing Cas9 to the *ori2* region. Surprisingly, we did not observe any cell killing as indicated by a high number of CFUs after induction of the CRISPR/Cas9 system, indicating that the gRNA binding sequence had already been eliminated. Upon further inspection, we found that the chromosomes were already fused while integrating the second homologous flank and therefore the sequence targeted by CRISPR/Cas9 was already lost. The obtained single chromosome strain was termed *V. natriegens* synSC1.0 and was verified after initial Sanger sequencing of the fusion sites by pulsed-field gel-electrophoresis (PFGE) and long-read whole-genome sequencing (Fig. 1B). The PFGE shows two bands of 1.9 and 3.2 Mb for the parental strain, which are absent in synSC1.0, showing only a single band with increased size of approx. 5.2 Mb. Long-read de novo assembly resulted in two circular contigs for the parental strain (3.2 and 1.9 Mb) and a single circular contig for synSC1.0 (5.2 Mb). Analysis of the chromosome fusion region in synSC1.0 revealed the absence of *dif1* and *ori2* as well as the expected chromosome fusion regions, despite a small deletion of 38 nucleotides corresponding to the ARNold^23^ predicted terminator of the deleted *rctB* gene, and was considered to be negligible (Fig. 1C).

### Fusing the two chromosomes of *V. natriegens* results only in minor growth differences

**Fig. 2:**
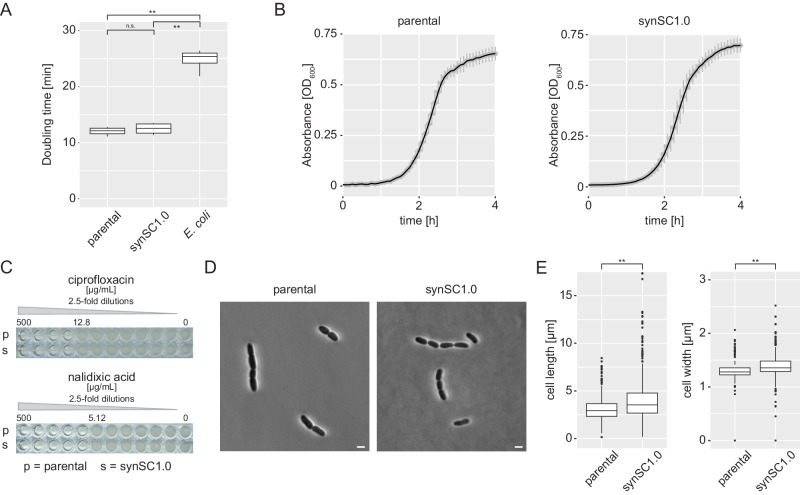
Comparative growth analysis of parental *V. natriegens* and synSC1.0. **A** Doubling times are determined to be 12 min 4 s (± 30.8 s) and 12 min 36 s (± 20.0 s) for the parental and synSC1.0 strain, respectively. The difference in growth rate is below 5%. *E. coli* was used as a control under the same conditions and doubling time was determined to be 24 min 48 s (± 34.7 s). Student’s *t*-test was applied to determine the significance; *p < 0.01, **p < 0.001, n.s. not significant. Experiments were performed in technical triplicates with biological quadruplicates. **B** Comparison of the parental and synSC1.0 growth curve do not reveal obvious differences. The growth curves show mean of biological quadruplicates, each consisting of three technical replicates. Standard deviation is indicated by gray bars. **C** MIC determination for ciprofloxacin and nalidixic acid for parental and synSC1.0. Both substances generate DNA replication stress by inhibiting gyrase function. No differences can be observed under the tested conditions. MIC tests were performed in quadruplicates and representative examples are shown, with the highest concentration allowing growth indicated. **D** Comparison of parental strain (left panel) and synSC1.0 (right panel) cells by microscopy. There were no drastic morphological differences for the average cells. A representative image in DIC is shown for both strains. Scale bar indicates 2 µm. **E** Comparative evaluation of cell length and cell diameter for the parental and synSC1.0 strain. Values were obtained using bacstalk from four biological replicates^55^; *n* = 663 cells (*n*_1_ = 32, *n*_2_ = 130, *n*_3_ = 213, *n*_4_ = 288) and *n* = 517 cells (*n*_1_ = 38, *n*_2_ = 103, *n*_3_ = 247, *n*_4_ = 129) for the parental and synSC1.0, respectively. The synSC1 strain shows on average a slightly increased cell size. Student’s *t-*test was applied to determine the significance; *p < 0.01, **p < 0.001, n.s. not significant.

To answer the most pressing question if the bipartite genome organization and the resulting time for DNA replication is the speed-limiting factor of *V. natriegens* rapid growth we performed comparative growth rate determination. We compared the growth of the parental strain with synSC1.0 and used *E. coli* MG1655 wild-type cells as an outgroup in LBv2 media^24^ (Fig. 2A). The minimal doubling time was determined to be 12 min 4 s (± 30.8 s) for the parental strain and 12 min 36 s (± 20.0 s) for synSC1.0 under our experimental conditions. The difference in growth is below 5% (statistically not significant), which is only a fraction of the expected 60% if replication would be the speed-limiting factor for *V. natriegens* rapid growth. This difference is lower compared to the observed generation time increase for the engineered *V. cholerae* MCH1, where an increase of 26% was observed^12^. A detailed analysis of the growth curves indicates no drastic alteration regarding lag-phase and total biomass under the tested growth conditions (Fig. 2B). In defined M9 media supplemented with 20.5 g/L NaCl and 0.4% glucose the doubling time is 22 min 46 s and 23 min 39 s for the parental and synSC1.0 strain, respectively (Fig. S2). Taking the increased time for DNA replication into account, we wondered if the parental strain would have an advantage under conditions causing replication stress. To test this, we assessed growth in the presence of ciprofloxacin or nalidixic acid, both gyrase inhibitors, initially in a minimum inhibitory concentration assay (MIC) (Fig. 2C) and subsequently for ciprofloxacin in MIC assays with fine adjusted concentrations (Fig. S3) but could not observe growth differences between the two strains. Further, we checked if synSC1.0 possesses an increased mutation rate in fluctuation assays but could not observe significant differences even in the presence of ethyl methanesulfonate (EMS) or methyl methanesulfonate (MMS), respectively. Suggesting DNA repair mechanisms are not impaired under the tested conditions (Fig. S4 and Table S1). To check whether an altered cell phenotype (e.g., elongated cell morphology) affected our optical density measurements, we performed light microscopy (Fig. 2D, E). No drastic differences were observed, which is consistent with the previously described rationally designed chromosome fusion of *V. cholerae* MCH1 with a single *ori*^12^ but not with the drastic phenotypic alteration for a natural single chromosome isolate of *V. cholerae* observed earlier^25^. This natural isolate possesses two *oris*, but *ori2* seems to be not functional in the natural isolate with a severe phenotype. Nevertheless, we observed individual aberrant cells in the synSC1.0 with a higher frequency compared to the parental strain (Fig. 2E, Fig. S5). The result may indicate an issue with chromosome segregation, cell division or chromosome dimer resolution at *dif2*. An increased number of chromosome dimers with increasing chromosome size was described previously in the study characterizing *V. cholerae* MCH1 using chromosome dimer resolution deficient mutants and could match the *dif* associated filamentation phenotype^12,19^. But it is more likely explained to be caused by cell division licensing problems caused by the nucleoid occlusion system as a consequence of SlmA misplacement on the chromosome, an observation made in MCH1^13,14^. However, taking our results together, the rational but drastic genome rearrangement does not cause major phenotypic alterations under the tested conditions.

### Replication pattern analysis indicates no differences

**Fig. 3:**
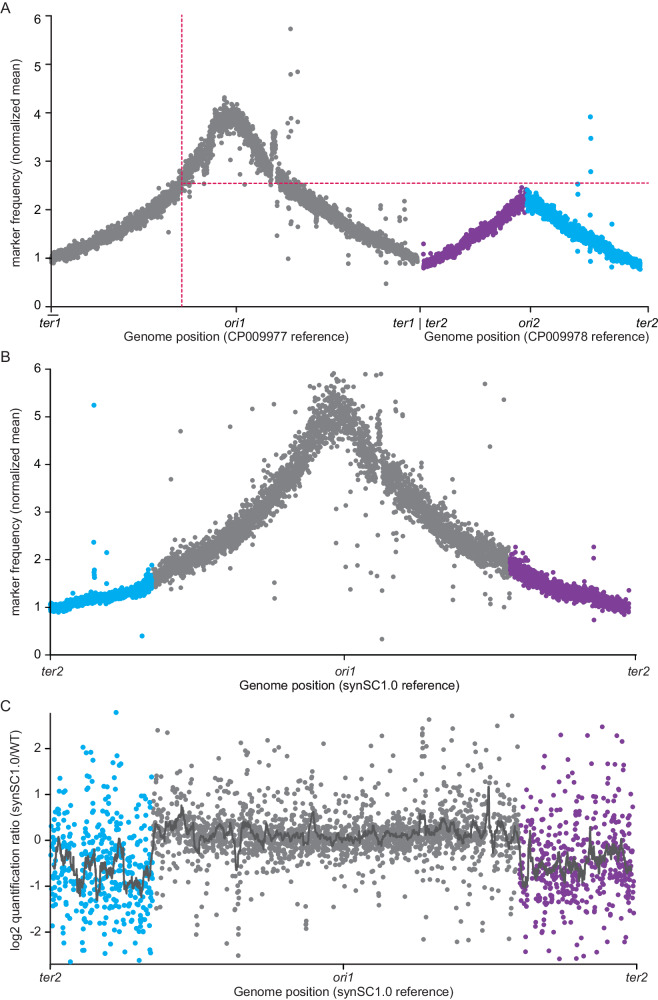
Comparative replication pattern analysis and global protein ratios of the parental *V. natriegens* and synSC1.0. **A**, **B** show relative read numbers for 1000 bp bins for the parental strain and synSC1.0, respectively. **A** Replication pattern analysis of the parental *V. natriegens* strain shows a single peak for each chromosome at the coordinates of the *ori1* and *ori2*. The relative copy number of *ori2* matches, as expected, to the relative copy number of location of the *crtS* site on chr1 indicated by red dotted lines. **B** Replication pattern analysis of synSC1.0 shows a single peak with its maximum at the *ori1* coordinates. These results confirm the fusion of the two chromosomes, the removal of *ori2*, and the absence of alternative or cryptic *oris*. Blue and purple indicate the chr2 halves according to the fusion site color code (*cf*. Fig. 1A). **C** Global protein abundance ratios for the parental and synSC1.0 strain. The plot shows a general higher protein abundance of chr1 (gray) and lower abundance of chr2 (blue and purple) encoded genes. Blue and purple indicate proteins expressed from chr2 halves according to the fusion site color code, and gray indicates proteins encoded on chr1. The gray line visualizes the moving window average of protein abundance in correlation to the genome sequence for a window of 50 kb with 1 kb steps. WT = parental strain.

To prove that the *ori2* was eliminated and no cryptic *ori* is responsible for the observed growth rate we performed replication pattern analysis of synSC1.0 in comparison to the parental strain (Fig. 3, Fig. S6). Genomic DNA of replicating cells and early stationary phase cells was extracted and submitted to whole-genome sequencing (Fig. S7). Replication pattern analysis of the parental strain shows the expected pattern for chr1 and chr2 with the highest marker frequency in the regions of *ori1* and *ori2* (Fig. 3A). The pattern of the data is consistent with the termination synchrony of the two chromosomes observed in *Vibrionaceae*^9^. A single peak in our replication pattern analysis of synSC1.0 verifies that the 5.2 Mb chromosome is replicated from *ori1* and no cryptic *ori* was formed (Fig. 3B). Notably the stationary phase cultures of synSC1.0 were not fully stationary in contrast to the parental strain and a fraction of cells were still replicating (Fig. S6). The *ori:ter* ratio based on the normalized data for the parental strain is approximately 4 and 2 for the two chromosomes, and the *ori:ter* ratio for synSC1.0 is approximately 5.5 (Fig. 3A, B). Note that the actual *ori:ter* ratio in synSC1.0 is slightly higher because the stationary phase samples were not fully stationary (Fig. S6) but were used to normalize the exponentially growing samples (Fig. 3B). Importantly, the *ori:ter* ratio of synSC1.0 roughly corresponds to the addition of the *ori:ter* of the two chromosomes of the parental strain. The DNA replication speed is not expected to be different in the two strains^26^. Based on the increased *ori:ter* ratio, we would expect an additional round of initiation of DNA replication resulting in potentially 32 replication origins within a cell; the documented maximum for *E. coli* is 16 origins under rapid growth conditions. However, our attempts to determine the number of replication forks using for *E. coli* established rifampicin/cephalexin replication run-out experiments based on flow cytometry did not work, which is consistent with reports for *V. cholerae*^27,28^. Alternative cell synchronization to determine the DNA content, such as activation of the stringent response by addition of the serine analog serine hydroxamate (SHX)^29^, was not attempted because it does not inhibit cell division in *V. cholerae* and most likely does not allow determination of the maximum DNA content under rapid growth conditions in *Vibrionaceae*.

The long- and short-read sequencing data of stationary phase samples were combined to perform a hybrid assembly to construct reference sequences for the parental and synSC1.0 strains with annotations based on the reference sequences as described in ref. ^18^. The resulting GenBank files are deposited within the NCBI BioProject PRJNA948340. The quantification data for the replication pattern analysis is provided in Supplementary Data S1.

### synSC1.0 shows an altered proteome composition as a result of the rational chromosome fusion

synSC1.0 does not show major phenotypic differences compared to the parental strain with respect to growth behavior and cell morphology. However, based on the global marker frequency change of the second chromosome (Fig. 3B), we would expect changes at the proteome level. We investigate global gene expression changes by whole proteome shotgun analysis for exponentially growing synSC1.0 compared to the parental strain. We find that proteins encoded by chr1 tend to be more abundant in synSC1.0, while proteins encoded by chr2 are less abundant compared to the parental strain (Fig. 3C and Fig. S8). This is expected based on the relatively reduced copy number of genes encoded by chr2 shown by the marker frequency analysis (see Figs. 3A, 3B, and S6). Alternatively, one could argue that the copy number of genes encoded by chr2 is not reduced, but the copy number of genes encoded by chr1 is increased during rapid growth. Almost all essential genes, and most of the genes required for rapid growth, are located on chr1^18^. We tested if the proteins encoded by genes previously classified as essential and growth-related are significantly more abundant compared to proteins encoded by all other genes. We found a small but significantly higher abundance of proteins required for rapid growth (Fig. S9). To further characterize synSC1.0 at the molecular level, we checked whether known *Kyoto Encyclopedia of Genes and Genomes* (KEGG) pathways were altered based on our proteomic data^30^. Proteins associated with processes related to rapid growth, such as genetic information processing, metabolism, and cellular processes, show a slight trend towards higher abundance in synSC1.0 (Fig. S10). However, the abundance of most proteins is not significantly different. Strikingly, the abundance of most proteins belonging to the KEGG pathway “*Replication and Repair*” are not significantly different, suggesting that the increased replisome size of synSC1.0 does not lead to DNA damage-related stress, which is consistent with the rest of the presented data. Therefore, our characterization of synSC1.0 shows that this strain may be a valuable strain for future biotechnological applications.

## Discussion

*V. natriegens* is the fastest growing bacterium known today and possesses a bipartite genome^16,17^. We fused the two chromosomes into a single chromosome replicated from a single *ori* and were expecting a strongly reduced growth rate for *V. natriegens*. However, the growth rate only slightly, but not significantly, deviates from that of the parental strain. This is in contrast to what was observed in *V. cholerae*, where a bigger difference was measured^12^. Recent literature indicates that there are potential challenges with cell division licensing in MCH1 presumable based on nucleoid occlusion system due to misplacement of the SlmA protein^13,14^. Nevertheless, we conclude based on our results that the growth rate of the parental *V. natriegens* is not limited by DNA replication because a 60% increased replicon size had only minor effects on the growth behavior. We assume an additional round of replication is initiated to compensate for the increased replicon size. However, we are not able to prove this experimentally but the replication pattern analysis hints into this direction.

It seems that our engineering approach does not disrupt the described replication-associated gene dosage relevant for the fast growth in *V. natriegens*, which is in contrast to what was described to be relevant for *V. cholerae* growth rate^7^. *V. cholerae* appears to be more sensitive to changes in gene dosage than *V. natriegens*. In particular, the expression of the translational machinery in the form of ribosomal operons has been shown to be important for growth and long-term evolution in *V. cholerae*^31–34^. Despite both species having a similar DNA replication progression^14^ the copy number alteration has a more severe impact on the growth of *V. cholerae*. Our conclusion and findings are consistent as described in ref. ^18^, which describes that chr2 content is less relevant to the rapid growth of *V. natriegens*. The gene dose of chr2 encoded genes under rapid growth conditions is reduced in synSC1.0 (Fig. 3B). This results in reduced expression of genes located on chr2, causing lower protein abundances (Fig. 3C and S8–10). The genes encoded on chr2 could be required for niche adaptation but seem to be dispensable for growth under rich or laboratory conditions. Our analysis of the proteome composition suggests that the reorganized chromosome configuration of synSC1.0 lowers expression of these dispensable genes, thereby liberating resources for growth-related processes like ribosomes and enzymes of central metabolism, which in itself could increase growth rate. The very minor decrease in growth rate of synSC1.0 compared to the parental strain could therefore be a combination of a beneficial effect of lowering expression of non-essential genes originally located on chr2 and the potentially detrimental effect of an increased replicon size.

The synSC1.0 strain characterized herein is a valuable resource to study chromosome biology in fast-growing bacteria. synSC1.0 allows for systematic genome engineering approaches by using the *ori2* region to build synthetic, single copy chromosomes and utilize the constructs as expression platforms or to relocate and isolate genes for distinct biological functions for their in-depth characterization^35–37^. Recently, developed genetic engineering tools such as NT-CRISPR, CRISPRi, and a reusable modular cloning part collection make this organism highly accessible^38–40^. Modular cloning approaches in combination with laboratory automation allow for rapid design-build-test-learn cycles using *V. natriegens* synSC1.0 with a reintroduced *ori2*-based synthetic chromosome as a platform for biotechnology and basic research questions^39,41–43^. Further, genome-scale modeling was established giving insights into *V. natriegens* metabolism and making this organism more predictable^44^. An additional advantage over the well-established *V. cholerae* model is the lack of pathogenicity allowing to conduct work in a biosafety level 1 environment, making synSC1.0 and its parental strain relevant to biotechnological and synthetic biology applications^17,24,45,46^. The halophilic nature of *V. natriegens* allows the use of seawater for its cultivation, making it a relevant emerging host in regard to a sustainable bioeconomy^47^.

## Material and methods

### Strains and growth conditions

*V. natriegens* was routinely grown in LB supplemented with v2 salts (204 mM NaCl, 4.2 mM KCl, and 23.14 mM MgCl_2_)^24^ or in M9 media supplemented with 20.5 g/L NaCl and the indicated carbon source. Chloramphenicol was added to a final concentration of 4 µg/mL for liquid and 2 µg/mL for solid medium if applicable. Standard *E. coli* laboratory strains were used for cloning, propagation, and archiving of plasmid DNA, all strains used and constructed in this study are provided in Table 1. Cultures were incubated at 37 °C and at 200 rpm in case of liquid cultures if not stated otherwise. Growth comparison of *E. coli* and *V. natriegens* was performed with v2 salt containing or NaCl supplemented media for all strains.

**Table 1:** Strains used and generated in this study

Name	Relevant features	Parental strain	Reference
*E. coli* MG1655	K-12 F^–^ λ^–^		^65^
*E. coli* NEB Turbo	K-12 *glnV44 thi-1* Δ(*lac-proAB*) *galE15 galK16 R*(*zgb-210*::Tn*10*)Tet^S^ *endA1 fhuA2* Δ*(mcrB-hsdSM)5*(r_K_^–^m_K_^–^) F′[*traD36 proAB*^+^ *lacI*^q^ *lacZΔM15*]		New England Biolabs (#C2984H)
*V. natriegens* Δ*dns*	Δ*dns*	*V. natriegens* ATCC14048	^39^
*V. natriegens*, DST026	Δ*dns with 3’ homology flank integrated between PN96_16275 and PN96_16280*	*V. natriegens* Δ*dns*	this study
*V. natriegens* synSC1.0	Δ*dns* Δ*dif1* Δ*ori2*	*V. natriegens*, DST026	this study

### Plasmids and oligonucleotides used in this study

Oligonucleotides were ordered and synthesized by Integrated DNA Technologies in 25 or 100 nM scale as standard desalted oligonucleotides (Table S2). All of the plasmids used in this study are listed in Table 2 and the plasmid files of the created plasmids are provided as GenBank files with Supplementary Data S2.

**Table 2:** Plasmids used and generated in this study

Name	Relevant features	Parental plasmid	Reference
pST_116 + 892/893	NT-CRISPR plasmid with gRNA for integration of 3’ homology flank. Created by oligo annealing of oDS_892 and oDS_893.	pST_116_LVL2 cam	^38^
pST_116 + 1420/1421	NT-CRISPR plasmid with gRNA for integration of 5’ homology flank. Created by oligo annealing of oDS_1420 and oDS_1421.	pST_116_LVL2 cam	^38^

### Genetic engineering of *V. natriegens*

tDNAs for integration of homology flanks through NT-CRISPR were generated by overlap extension PCR^48^. Sequences of tDNAs are provided in Supplementary Data S2 as GenBank files. The construction of NT-CRISPR plasmids with gRNAs targeting the integration sites were constructed as described previously through annealing of oligonucleotides^38^. Annealing reactions were setup by mixing 1.5 μL of each oligonucleotide (100 μM) with 5 μL T4-DNA ligase buffer (Thermo Scientific) in a total reaction volume of 50 μL. Reactions are incubated in a heat block at 95 °C for 15 min, before switching off the heat block for slowly cooling down the samples to room temperature (~1 h). Cloning reaction with the NT-CRISPR plasmids was setup with ~200 ng of the respective plasmid, 3 μL annealed oligonucleotides, 0.5 μL of T4-DNA Ligase (5 Weiss U/μL, Thermo Scientific) and BsaI (10 U/μL) and 1 μL T4-DNA ligase buffer in a total reaction volume of 10 μL. Reactions were run in a thermocycler with 30 cycles of 37 °C (2 min) and 16 °C (5 min), followed by a final digestion step at 37 °C for 30 min and an enzyme denaturation step at 80 °C for 10 min.

The integration of the homologous flanks were performed as described for the NT-CRISPR method^38^. tDNAs consisted of 3 kb homologous flanks and 3 kb insert sequence. The insert sequence is identical to the sequence upstream and downstream of *dif1* and enable fusion of chromosomes. At first, we integrated the 3′ homology flank and subsequently the 5′ homology flank. Successful integration of the 3′ homology flank was confirmed by cPCR with oligonucleotides oDS_920 and oDS_921. Integration of 5′ homology flanks and the spontaneous fusion of both chromosomes was confirmed with the primer pairs oDS_914/oDS_915 and oDS_916/oDS_917. Generation of a PCR fragment for both primer pairs indicates successful integration of 5′ homology flank without chromosome fusion, while the absence of a band for oDS_916/oDS_917 indicates chromosome fusion. Chromosome fusion was subsequently verified through Sanger sequencing (Microsynth Seqlab). Two PCR fragments spanning the junctions were generated with the primer pairs oDS_1462/oDS_1464 and with oDS_1460 and oDS_1461, each amplicon was sequenced with two reactions with primers oDS_1463/oDS_1477 and oDS_1455/oDS_1459, respectively.

### Pulsed-field-gel-electrophoresis

Plug preparation for yeast standards was performed according to described methods by Hage and Houseley^49^. Bacterial plug preparation was performed similar, with the following alterations: Cultures were grown overnight at 30 °C with 200 rpm. An equivalent of 1 mL OD_600_ = 5 was harvested and used for plug preparation. The concentration of low melting agarose (SeaKem LE, Lonza) for plug preparation was reduced to 0.8%, and lysozyme (1 mg/mL) was used instead of lyticase. PFGE was undertaken by running samples on a 0.8% agarose gel using Pulsed-Field Certified Agarose (Bio-Rad) in 1× TAE buffer at 14 °C on a Bio-Rad clamped homogeneous electric field apparatus (CHEF-DR III, Bio-Rad). 3 V/cm were used with 46 h switch time of 600 s at 120 °C. The resulting gel was stained with 1× SYBR Safe (ThermoFisher Scientific) and imaged using Typhoon RGB laser scanning system. The known karyotypes of *S. cerevisiae* and *S. pombe* served as size standards.

### Nanopore sequencing and data analysis

*V. natriegens* strains were cultured in 10 mL LBv2 overnight. DNA was extracted using the Monarch Genomic DNA Purification Kit (NEB) according to the manufacturer guidelines. Each sample was split in 4 purifications which were pooled subsequently again. 2 µg gDNA, corresponding to a 5-fold increase to the recommended input DNA was used as input for the library preparation using the SQK-LSK109 kit; the reason for the increase was the use of approx. 50 kb high molecular weight DNA compared to the 10 kb sized input DNA according to the protocol. The remaining procedure was performed according to the manufacturer guidelines. Each sample was sequenced on a single Flongle flow cell (FLO-FLG001 (R9.4.1)). Basecalling of raw sequencing data was performed utilizing guppy (version 6.4.6+ae70e8f; Oxford Nanopore Technologies). Basecalled raw data are deposited in BioProject PRJNA948340 individual accession IDs are provided in Table S3. Initial de novo assembly was performed with canu (version 2.2)^50^ resulting in two and one circular chromosomes for the parental and synSC1.0 strains respectively. Dot blots of de novo assemblies in comparison to the corresponding references based on *V. natriegens* ATCC14048 reference sequences (CP009977 and CP009978) and the in silico designed single chromosome reference were performed using mummer (version: 3.5)^51^. Analysis of dot blots indicated duplicated segments which were later identified as assembly artifacts and were the reason to generate reference sequences by combining long and short-read sequencing data of this study (*cf*. section “*Hybrid assembly and reference construction*”).

### Plate reader-based growth assays

Plate reader-based growth assays were adjusted to *V. natriegens* based on our previously published procedure^52,53^. Briefly, *V. natriegens* precultures were inoculated from a single colony and grown for 6 hours in LBv2 at 37 °C with 200 rpm. Cultures were arranged in microtiter plates and subsequently inoculated into clear, flat bottom microtiter plates (#655185, Greiner Bio-One GmbH) using a Rotor HDA+ screening robot (Singer Instruments) containing the indicated media and supplements. Plates were sealed using a PlateLoc plate sealer (Agilent) with optical clear seal. Growth was monitored in ClarioStar Plus plate readers (BMG) equipped with specific plate holders for extensive kinetics under shaking conditions. Different settings were extensively tested prior the following settings were identified to be the best conditions for *V. natriegens* with our setup and used throughout the study: 2 min linear shaking prior OD_600_ measurement, 800 rpm orbital shaking during idle time at 37 °C, cycle time was set to 5 min and kinetic was monitored for up to 24 h. Raw data was exported and analyzed in R with the growthcurver package (v0.3.1)^54^. All experiments were performed in biological quadruplicates each with technical triplicates. *E. coli* MG1655 served as an external control.

### Minimum inhibitory concentration (MIC) assay

MIC tests were performed as kinetic in ClarioStar Plus plate readers (BMG) with the settings described above for plate reader-based growth assays. The only alteration was the preparation of the microtiter plate where the broth dilution method was used to determine the MIC as described previously^52^. The rationale behind this procedure was to be able to analyze growth in detail in contrast to only perform an endpoint measurement. In addition, microtiter plates were scanned at the end of the assay using an Epson Perfection V700 Photo scanner. All experiments were performed in biological quadruplicates.

### Rifampicin fluctuation assay to determine mutation frequency

Bacterial cultures were grown overnight from a single colony. 3 mL of LBv2 media without substance or test conditions (EMS [1:1,000](Sigma–Aldrich, #M0880) or MMS [1:10,000](Sigma–Aldrich, #129925)) were inoculated 1:1,000 and grown for 6 h at 37 °C with 200 rpm, respectively. 100 µL of respective dilutions were plated onto LBv2 media (10^−6^–10^−^^8^) with and without 50 µg/mL rifampicin (10^0^–10^−^^1^). The mutation frequency was determined based on CFUs (Table S1 and Fig. S4). All experiments were performed in biological quadruplicates.

### Microscopic imaging and analysis

*V. natriegens* precultures were grown overnight from a single colony, inoculated 1:100 in LBv2 media, and grown at 37 °C with 200 rpm for 1.5 h. 1.5 µL of exponential phase cultures were immobilized on 2% low gelling agarose (Sigma) pads containing LBv2 media and analyzed using an Axioplan 2 phase contrast microscope (Zeiss) and a Plan Neofluar 100× objective (Zeiss). Extraction of cell length and width was performed using bacstalk^55^.

### Replication pattern analysis

Cultures for extraction of genomic DNA for replication pattern analysis were started from an overnight culture in 5 mL LBv2, (16 h, 37 °C, 200 rpm) to an OD_600_ of 0.001 in 100 mL in 1 L baffled shake flasks. Samples for the exponential phase were taken after approximately 2 h (OD_600_ ≈ 0.3). Samples for stationary phase were taken after 12 h (OD_600_ ≈ 10). A culture volume equivalent to 1 mL of OD_600_ = 2 was harvested by centrifugation for 1 min at 20,000 × *g* at 4 °C. The same cultures were used to obtain cell material for whole proteome analysis (see section “Shotgun proteomics analysis” for details). Supernatant was discarded and pellet was stored at −80 °C. DNA was extracted using the Monarch Genomic DNA Purification Kit (NEB) according to the manufacturer guidelines. Library generation and short-read sequencing was performed by an external service provider with a PCR-free 150 paired-end sequencing workflow (Novogene). Replication pattern analysis was performed with Repliscope (v1.1.1)^56^. BED files containing the number of reads per 1 kb bin were generated using the localMapper within the Repliscope software. The BED files were used to plot the data using R. Sample normalization for the parental and synSC1.0 strain were performed using the mean bin value of 25 kb of the left and right terminus regions of the *ori*-centered references for chr1 (CP009977) and synSC1.0, respectively. Averaged of the three replicates were generated and used to plot the data. The values for each bin, normalized values, mean, and standard deviation are provided in Supplementary Data S1. All short-read sequencing raw data are deposited in BioProject PRJNA948340 and individual accession IDs are provided in Table S3.

### Hybrid assembly and reference construction

Flye (version 2.9.1-b1780) was used for de novo assembly of long-reads^57^. Resulting assemblies were corrected against the corresponding references using RagTag (version 2.1.0)^58^, respectively; *V. natriegens* ATCC14048 (CP009977 and CP009978)^18^; *V. natriegens* synSC1.0 (in silico designed based on CP009977 and CP009978). Polypolish (version 0.5.0) was used with standard settings to obtain polished reference genomes for both strains using short-reads from stationary phase samples (Tab. S2)^59^. Validation and quality assessment of the assemblies was performed using Quast (version 5.2.0)^60^. The origin of replication of each reference were set to nucleotide +1, resulting references are deposited within the BioProject PRJNA948340 (Table S2).

### Shotgun proteomics analysis

For whole proteome analysis, cell material equivalent to 6 mL at OD_600_ = 0.5 was collected, washed twice in PBS (pH = 7.4), and stored at -80 °C. Notably, the samples are taken from the same cultures in which the replication pattern analysis was performed. Cells pellets were resuspended in 300 μl lysis buffer (2% sodium lauroyl sarcosinate (SLS), 100 mM ammonium bicarbonate) and heated for 10 min at 90 °C. The amount of proteins was determined by bicinchoninic acid protein assay (Thermo Scientific). Proteins were reduced with 5 mM Tris(2-carboxyethyl) phosphine (Thermo Fischer Scientific) at 90 °C for 15 min and alkylated using 10 mM iodoacetamide (Sigma–Aldrich) at 20 °C for 30 min in the dark. For tryptic digestion 50 µg protein was incubated in 0.5% SLS and 1 µg of trypsin (Serva) at 30 °C overnight. Following digestion, the SLS was precipitated by adding acid, and tryptic peptides were desalted using C18 solid phase extraction cartridges (Macherey-Nagel).

Dried peptides were reconstituted in 0.1% Trifluoroacetic acid and then analyzed using liquid-chromatography-mass spectrometry carried out on a Exploris 480 instrument connected to an Ultimate 3000 RSLC nano and a nanospray flex ion source (all Thermo Scientific). Peptide separation was performed on a reverse phase HPLC column (75 μm × 42 cm) packed in-house with C18 resin (2.4 μm; Dr. Maisch). The following separating gradient was used: 94% solvent A (0.15% formic acid) and 6% solvent B (99.85% acetonitrile, 0.15% formic acid) to 25% solvent B over 40 min, and an additional increase to a final of 35% solvent B over 20 min at a flow rate of 300 nl/min.

MS raw data was acquired on an Exploris 480 (Thermo Scientific) in data-independent acquisition (DIA) mode. All MS acquisition parameters are described in ref. ^52^. Analysis of DIA data was performed using the DIA-NN version 1.8^61^ using a protein database from *Vibrio natrigens* based on BioProject: PRJNA267132^18^ to build a dataset-specific spectral library for DIA-NN analysis.

The neural network-based DIA-NN suite performed noise interference correction (mass correction, RT prediction, and precursor/fragment co-elution correlation) and peptide precursor signal extraction of the DIA-NN raw data. The following parameters were used:

Full tryptic digest was allowed with two missed cleavage sites, and oxidized methionines and carbamidomethylated cysteines. “Match between runs” and “remove likely interferences” were enabled. The neural network classifier was set to the “single-pass mode”. Quantification strategy was set to any LC (high accuracy). Cross-run normalization was set to RT-dependent. Library generation was set to smart profiling. DIA-NN outputs were further evaluated using the SafeQuant^62,63^ script modified to process DIA-NN outputs.

### Statistics and reproducibility

Sample size and number of replicates are stated for each experiment. Generally, four biological replicates were performed with three technical replicates if not stated otherwise. Student’s *t*-test (unpaired, two-tailed) was applied to indicate significance. Statistical analysis was performed using R/RStudio (https://www.r-project.org/ and https://posit.co/).

### Reporting summary

Further information on research design is available in the Nature Portfolio Reporting Summary linked to this article.

### Supplementary information

Supplementary Information

Description of Additional Supplementary Files

Supplementary Data S1

Supplementary Data S2

Supplementary Data S3

Supplementary Data S4

Reporting Summary

## Data Availability

The data underlying this study are available in the published article and its online supplementary material. Sequencing raw reads and constructed reference sequences are deposited at NCBI under BioProject PRJNA948340. The mass spectrometry proteomics data have been deposited to the ProteomeXchange Consortium via the PRIDE^64^ partner repository with the dataset identifier PXD049476 and quantification is provided in Supplementary Data S3. All growth and microscopy-related numerical data is provided in Supplementary Data S4. Microscopy and pulsed-field gel-electrophoresis raw data are deposited at Edmond the Max Planck Society Repository and can be accessed at the following doi: 10.17617/3.BRUKUO. All material created within this study is available from the corresponding author upon request.
