# Supplementary material for: Rationally designed chromosome fusion does not prevent rapid growth of Vibrio natriegens
Source: Commun Biol. 2024 May 2;7:519. doi: 10.1038/s42003-024-06234-1 (PMC11066055; doi:10.1038/s42003-024-06234-1)
Supplement: Supplementary file 1 — Supplementary Information [file 42003_2024_6234_MOESM1_ESM.pdf]

Supplementary Information

Rationally designed chromosome fusion does not prevent rapid growth of *Vibrio natriegens*

Authors: Lea Ramming[1,#], Daniel Stukenberg[2,#], María del Carmen Sánchez Olmos[1], Timo Glatter[1], Anke Becker[2,3], Daniel Schindler[1,3,*]

Affiliations

[1] Max Planck Institute for Terrestrial Microbiology, Marburg, Germany

[2] Department of Biology, Philipps-Universität Marburg, Marburg, Germany

[3] Center for Synthetic Microbiology, Philipps-Universität Marburg, Marburg, Germany

# These authors contributed equally

*** correspondence to:** daniel.schindler@mpi-marburg.mpg.de

This file contains:

Figure S1-S10

Table S1-S3

Supplementary References

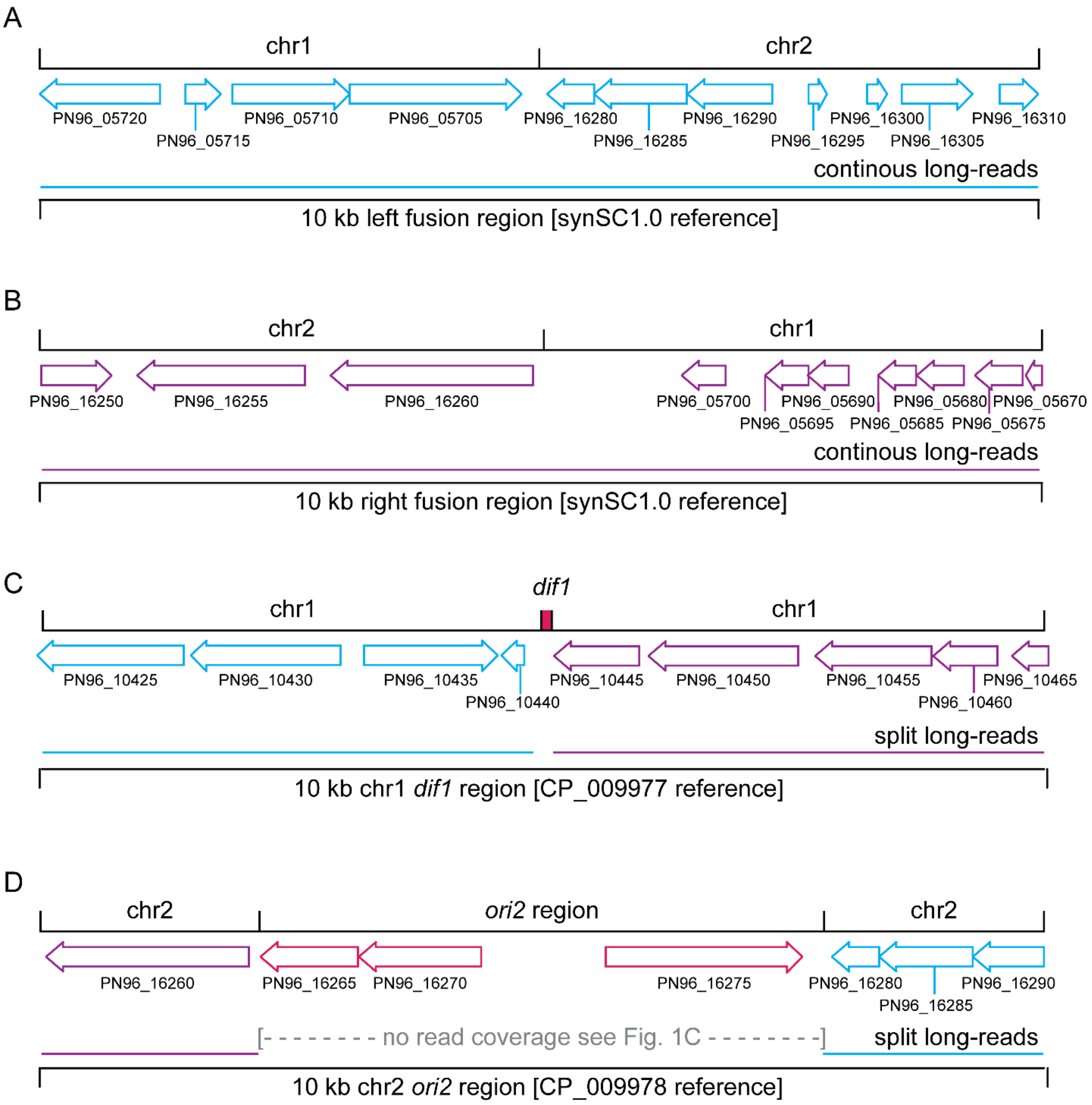

Figure S1 | Detailed annotation of genomic regions visualized in Figure 1C. Annotations are performed according to GenBank files CP009977 (chr1) and CP009978 (chr2) with their respective gene annotations. Purple and blue colors match the homology regions depicted in Figure 1A for the fusion of the two chromosomes. Red colors indicate the deletion of the *ori2* region consisting of the *parAB* operon (PN96_16270 and PN96_16265) and *rctB* (PN96_16270). The scale of the genomic region is 10 kb.

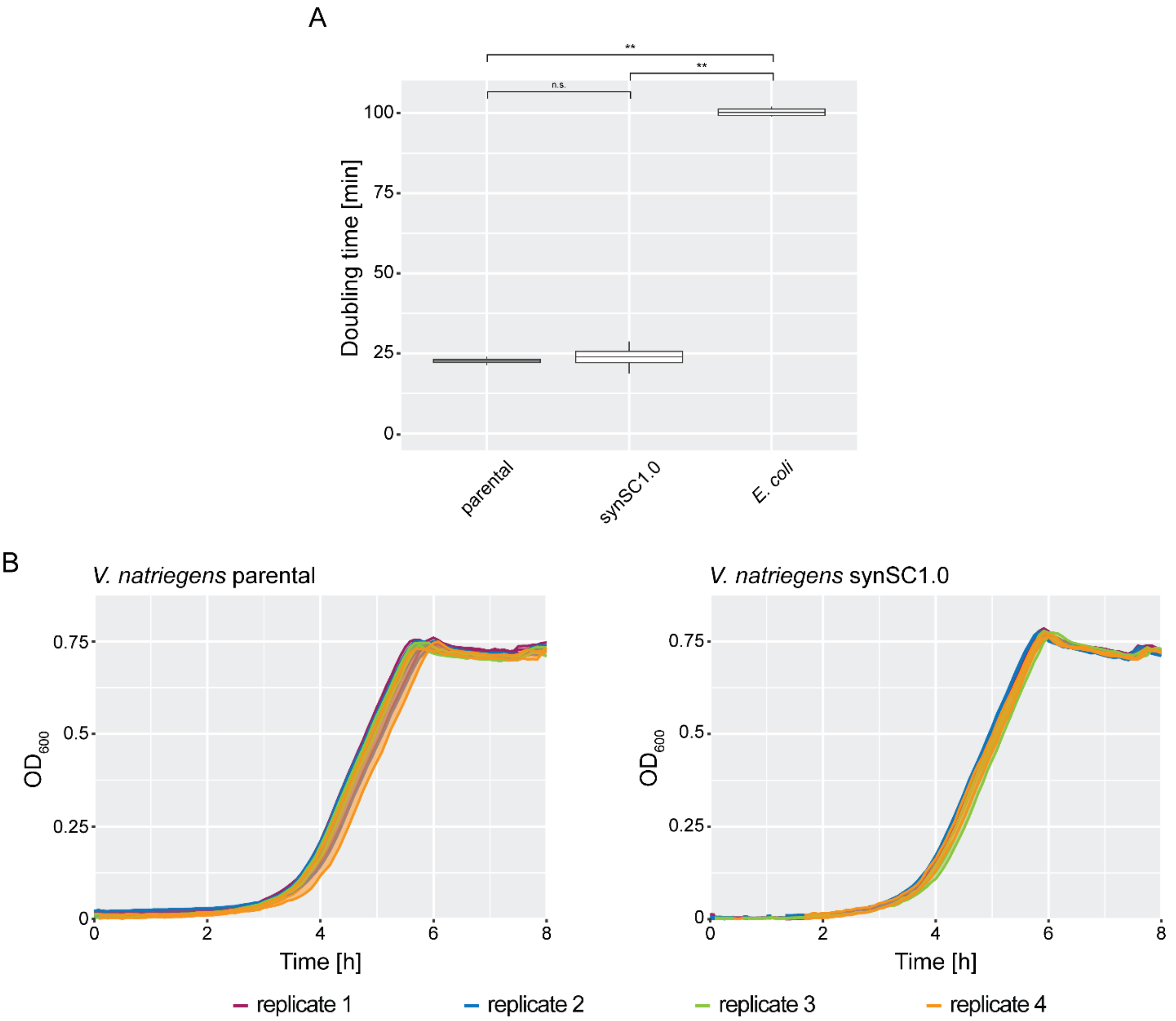

Figure S2 | Comparative growth assay parental strain and synSC1.0 in M9 media supplemented with 20.5 g/L NaCl and 0.4% glucose. (A) Doubling times are determined to be 22 min 46 s (+/- 43.8 s) and 23 min 39 s (+/- 24.6 s) for the parental and synSC1.0 strain, respectively. The difference in growth rate is 4.1%. *E. coli* was used as a control under the same conditions and doubling time was determined to be 1 h 40 min 12 s (+/- 3 min 20.8 s). However, the conditions presumably cause high salt stress for *E. coli*. Student's t-test was applied to determine the significance; *: p< 0.01, **: p<0.001, n.s. not significant. Experiments were performed biological quadruplicates each with technical triplicates. (B) Growth curve of *V. natriegens* parental (left panel) and synSC1.0 (right panel). We observed no extended lag-phase for synSC1.0 in repeated experiments from dense overnight cultures. Previously, we observed shorter lag-phase for synSC1.0 which may be explained by at least a fraction of cells still replication (see Figure S6D-E for replication pattern details).

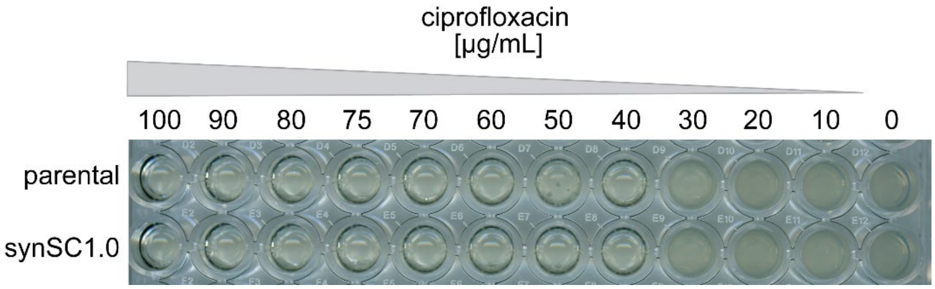

Figure S3 | MIC assay for ciprofloxacin with smaller dilution steps to compare the parental and synSC1.0 strain. No differences are observed in four biological replicates. A representative scan is shown.

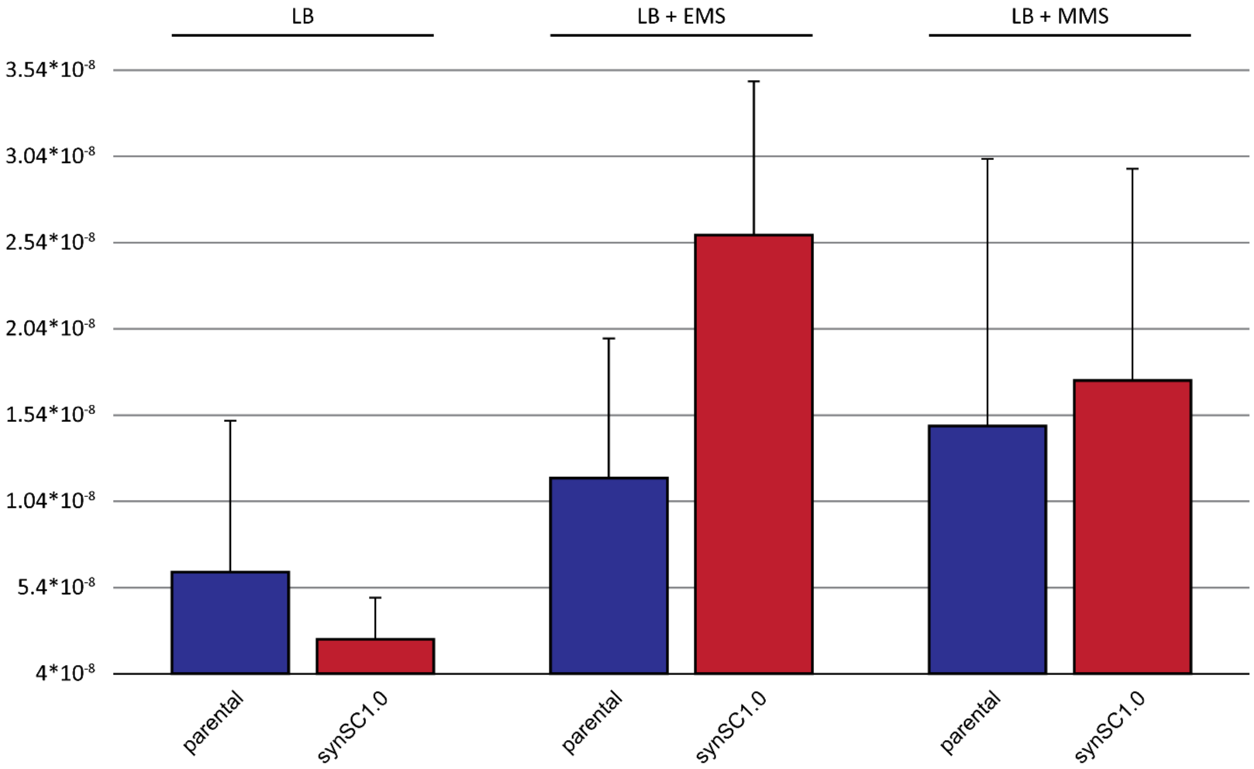

Figure S4 | Comparative fluctuation assay of the parental strain and synSC1.0. No drastic differences were observed between the two strains which is consistent with the MIC test and other characterization experiments (see Figure 2). Experiments were performed in biological quadruplicates, CFU values are provided in Table S1. Error bars indicate standard deviation.

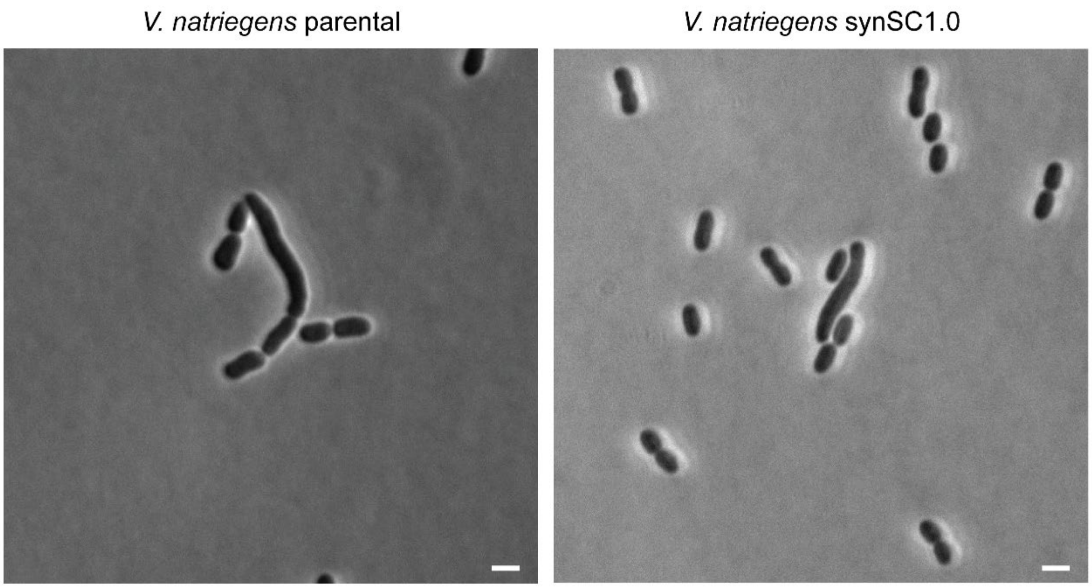

Figure S5 | Elongated cell phenotype observed for *V. natriegens* under rapid growth conditions. Elongated cells were sporadic observed in both the parental and synSC1.0 strain. Representative image for the parental (left panel) and synSC1.0 (right panel) are shown. The observed phenotype was more frequent for synSC1.0 (*cf*. Figure 2F). It may be the result of reduced chromosome dimer resolution fidelity previously observed in *V. cholerae*[1]. More severe phenotypes affecting the whole cell population were described earlier for a natural isolate with fused chromosomes of *V. cholerae* but are not observed for synSC1.0[2]. However, recent literature indicates that there are potential challenges with cell division licensing in MCH1 presumable based on nucleoid occlusion system due to misplacement of the SlmA protein[3,4]. Scale bar 2 µm.

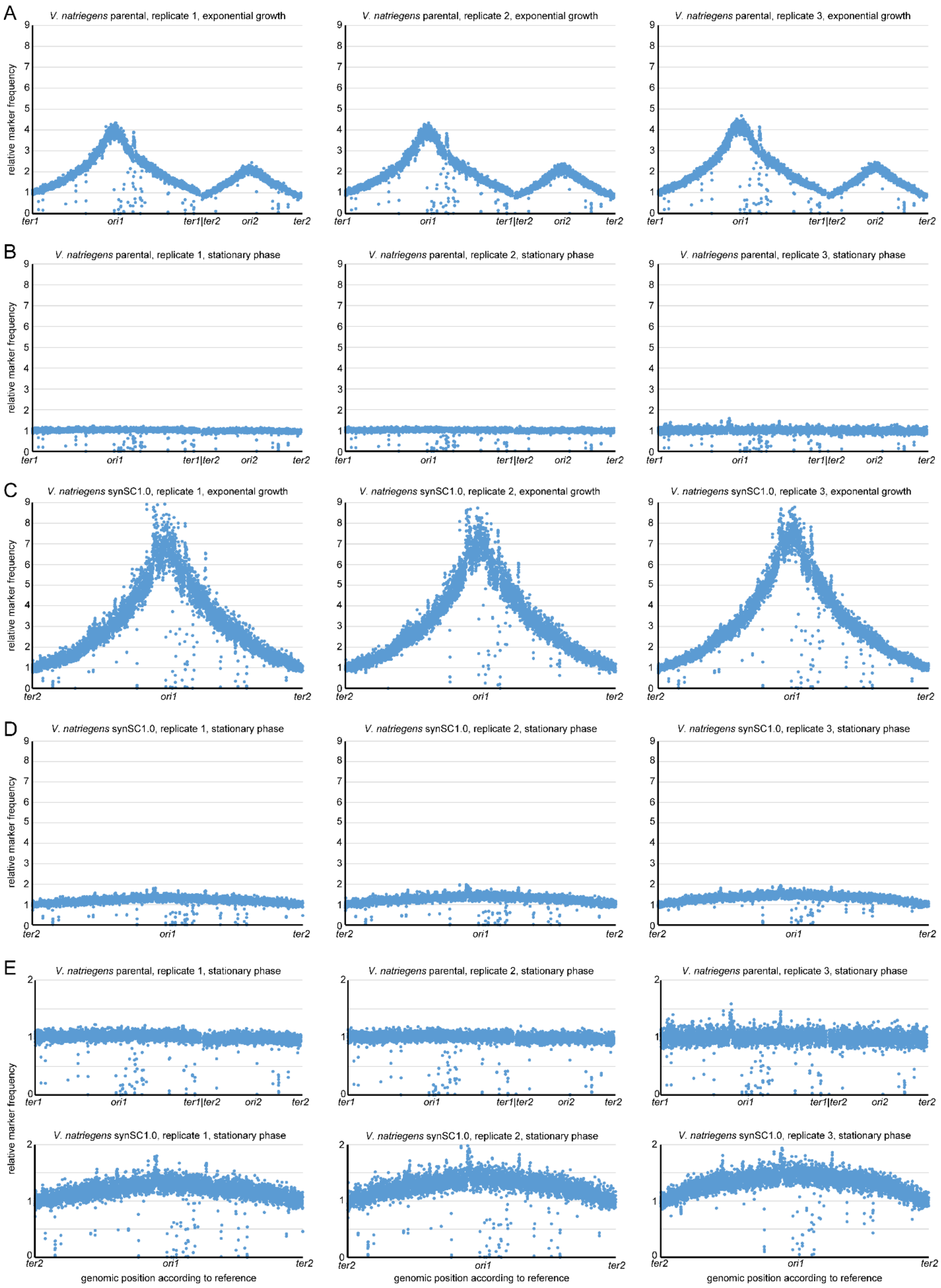

[Figure legend on the next page]

Figure S6 | Coverage plots of samples for replication pattern analysis based on normalized bin read count for each sample. Normalized marker frequency analysis (1000 bp bins) for (A) the *V. natriegens* parental strain in exponential growth phase, (B) early stationary phase, (C) the *V. natriegens* synSC1.0 strain in exponential growth phase and (D) in early stationary phase respectively. (E) Visualizes a magnification of (B) and (D) for each replicate indicating that a fraction of *V. natriegens* synSC1.0 cells in the stationary phase are still replicating DNA. This observation is unexpected because the samples were in regard to their optical density measurements in stationary phase. The samples were taken at a later time point compared to the initial study because this effect was observed earlier[5]. This indicates that the *ori*:*ter* ratio of synSC1.0 is higher compared to the plotted value in Figure 3B because in the normalization step where the ratio of replicating/non-replicating data is generated the ratio obtained is reduced because a fraction of cells is still finishing DNA replication. This might be an explanation why the *V. natriegens* synSC1.0 cells have a shorter lag phase in minimal media (Figure S2). All plots were generated based on the BED files generated with Repliscope[6]. Data was normalized and plotted using R for details see Material and Methods. Data is available in Supplementary Data S2. The same samples were used to analyze the relative protein abundances (Figure 3C).

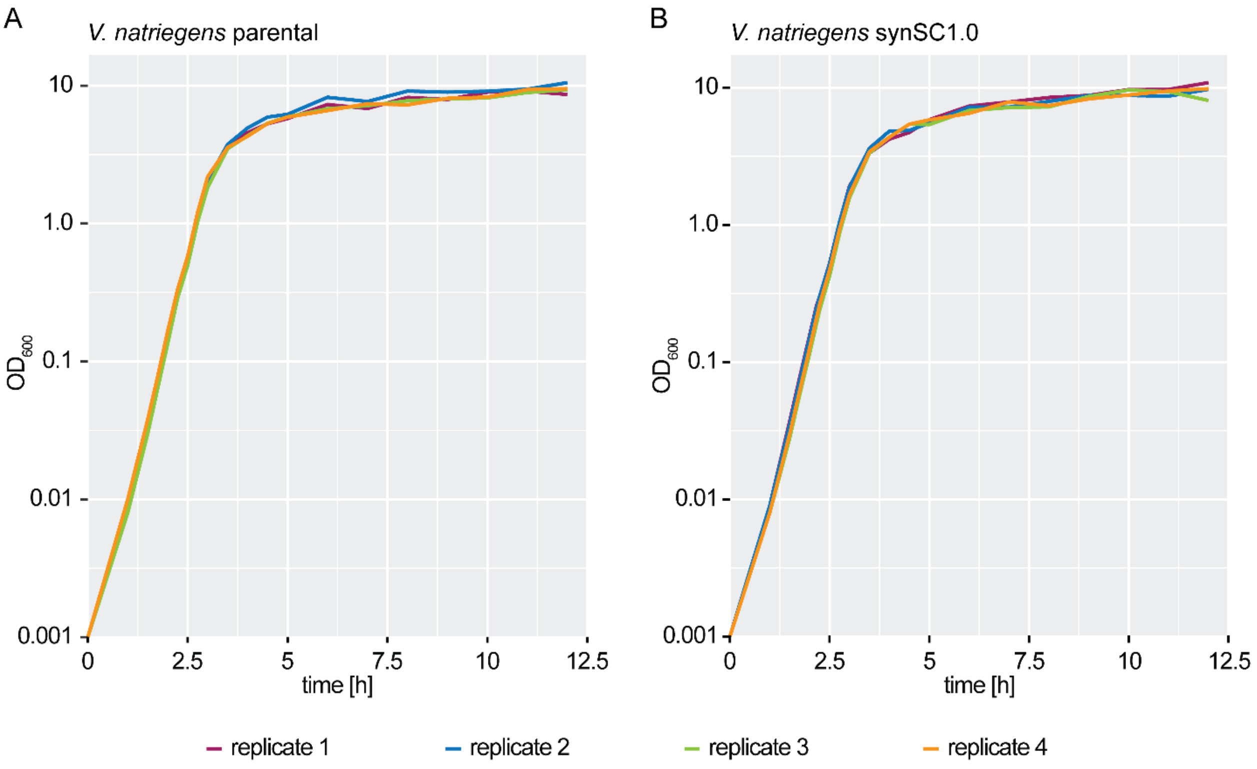

Figure S7 | Growth curves of replicates for replication pattern and shotgun proteomics samples. Growth curve data of replicates of (A) the parental strain and (B) the synSC1.0 strain. The color of each replicate is identical in both plots and visualized in purple, blue, green and orange. Replicates one to three are used for replication pattern analysis (Figure 3A-B and S6) and replicates one to four are used for shotgun proteomics (Figure 3C and S8). OD values are plotted to a log10 axis. Exponential growth phase samples for replication pattern analysis and shotgun proteomics were harvested at $OD_{600} \approx 0.3$. Stationary phase samples for replication pattern analysis at after 12 hours at $OD_{600} \approx 10$.

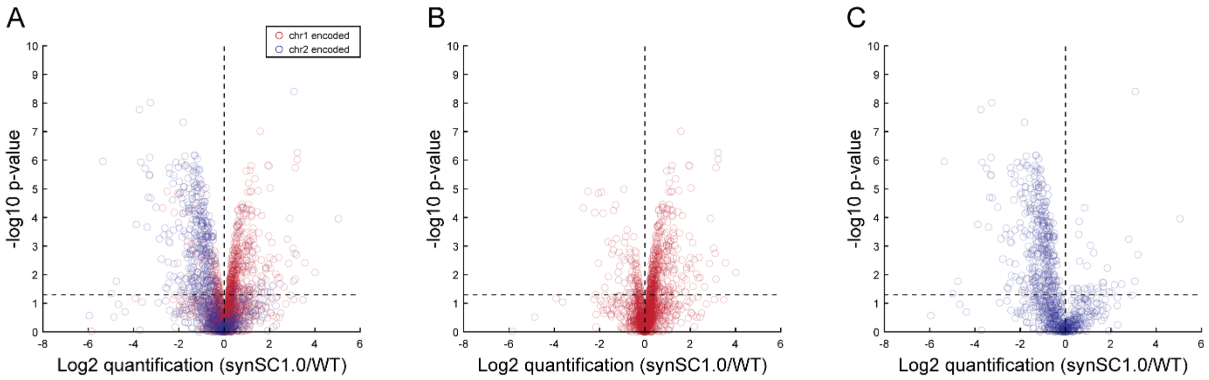

Figure S8 | Volcano plots of shotgun proteome analysis. (A) Visualizes the differential protein abundance for chr1 (red) and chr2 (blue) encoded gene products. (B) and (C) visualize chr1 and chr2 separately. The results show that the altered marker frequency based on the chromosomal fusion leads to a higher abundance of proteins encoded in chr1 and to a reduction of chr2 encoded proteins. WT = parental strain.

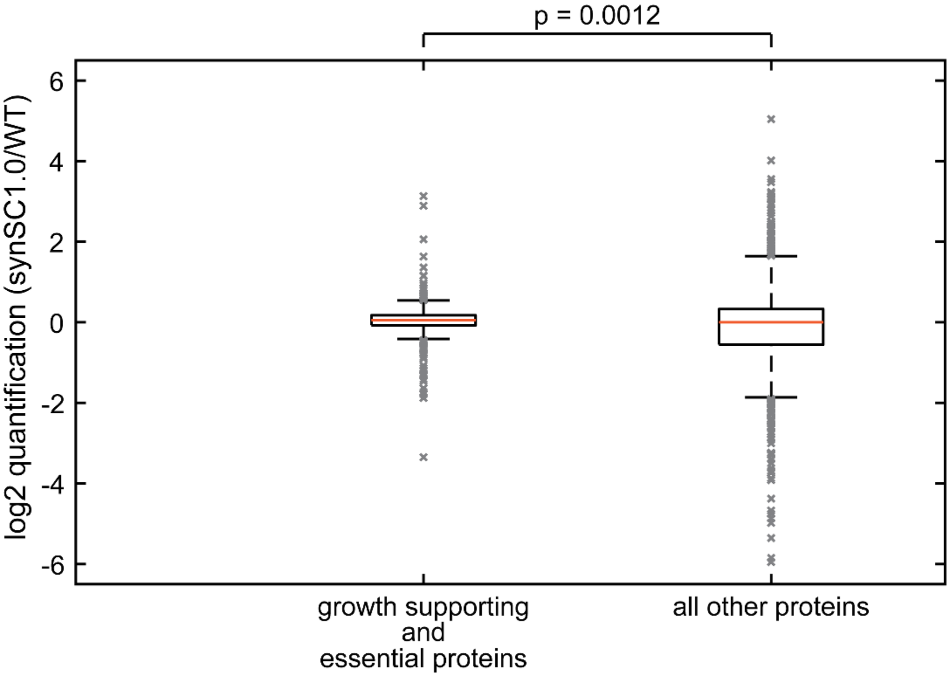

Figure S9 | Comparison of protein intensity ratios between synSC1.0 and the parental strain for genes required for rapid growth and all other genes. Classification of genes and associated proteins was based on the CRISPRi screen by Lee et al. 2019[7]. Data for genes with a significant beta score in LB3 are shown in the left boxplot (n = 584). Data for all other genes boxplot are shown in the right boxplot (n = 2276). P-value was calculated with a two-sample t-test.

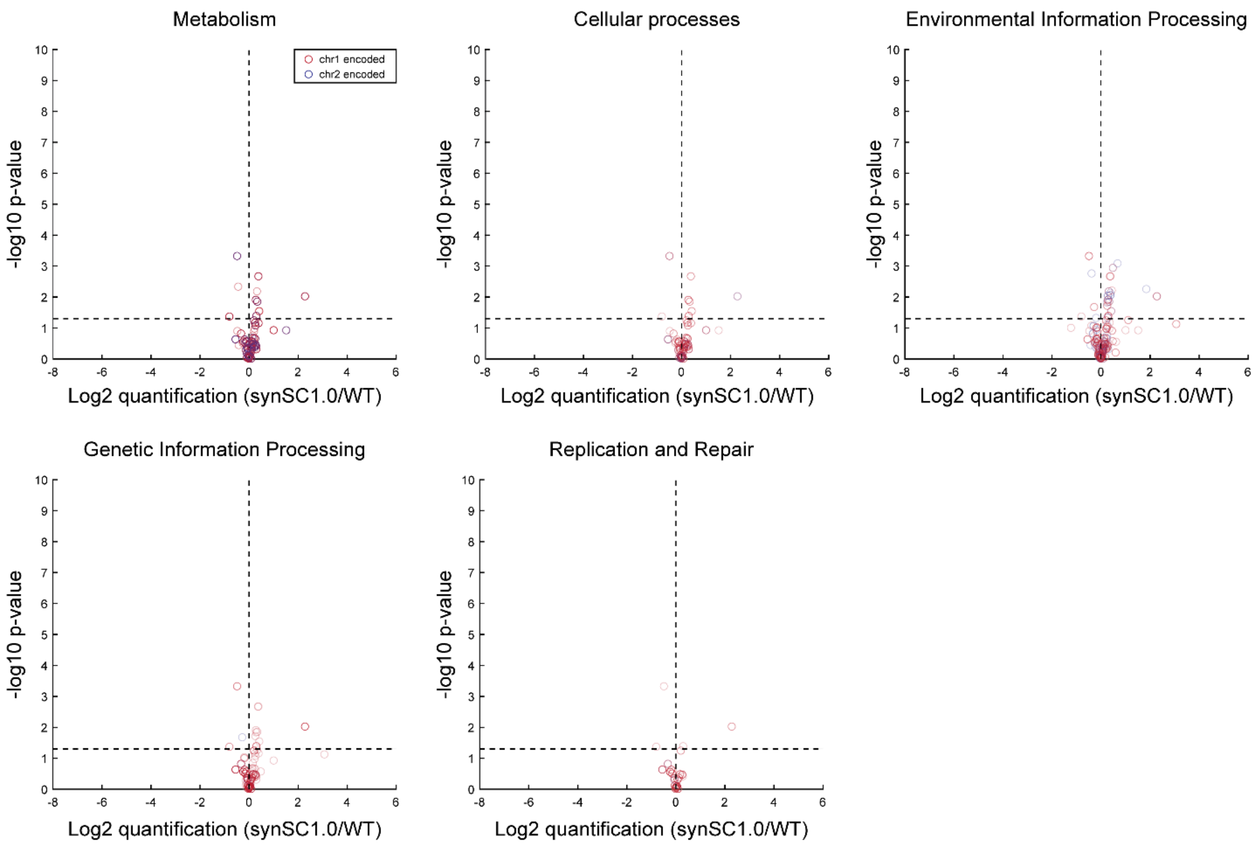

Figure S10 | Volcano plots of proteome data based on selected KEGG pathway enrichments. No drastic alterations of protein levels can be observed for core pathways of *V. natriegens* synSC1.0. Strikingly, DNA replication and repair does not show a global alteration matching the data of the other synSC1.0 characterization experiments. WT = parental strain.

Table S1 | Fluctuation assay CFU counts. Undil. = undiluted, nc = non-countable, R1 to R4 = replicates

V. natriegens parental													
**Medium**	**Dilution**	**LB [control]**				**LB + EMS**				**LB + MMS**			
		**R1**	**R2**	**R3**	**R4**	**R1**	**R2**	**R3**	**R4**	**R1**	**R2**	**R3**	**R4**
**LB**	**10^{-6}**	640	404	420	276	506	568	620	856	244	404	292	232
**LB**	**10^{-7}**	110	47	222	38	nc	160	83	104	49	141	55	68
**LB**	**10^{-8}**	14	4	107	2	13	10	8	22	46	49	nc	22
**LB Rif**	**undil.**	5	3	0	1	3	9	4	15	25	4	21	8
**LB Rif**	**10^{-1}**	0	0	0	1	1	0	3	2	2	0	1	1
**LB mean**		1047	425	4447	285	903	1056	750	1365	1778	2238	421	1037
**LB Rif mean**		2.5	1.5	0	5.5	6.5	4.5	17	17.5	22.5	2	15.5	9
**Ratio [LB Rif/LB]**		2.4 $*10^{-9}$	3.5 $*10^{-9}$	0	1.9 $*10^{-8}$	7.2 $*10^{-9}$	4.3 $*10^{-9}$	2.3 $*10^{-8}$	1.3 $*10^{-8}$	1.3 $*10^{-8}$	8.9 $*10^{-10}$	3.7 $*10^{-8}$	8.7 $*10^{-9}$
**Mean ratio**		**6.3 $* 10^{-9}$**				**1.2 $* 10^{-8}$**				**1.5 $* 10^{-8}$**			

V. natriegens synSC1.0													
**Medium**	**Dilution**	**LB [control]**				**LB + EMS**				**LB + MMS**			
		**R1**	**R2**	**R3**	**R4**	**R1**	**R2**	**R3**	**R4**	**R1**	**R2**	**R3**	**R4**
**LB**	**10^{-6}**	500	336	412	336	332	316	324	296	28	32	105	58
**LB**	**10^{-7}**	50	12	50	49	18	60	26	18	2	9	19	36
**LB**	**10^{-8}**	7	12	8	9	5	4	5	6	0	1	2	4
**LB Rif**	**undil.**	0	1	6	4	2	22	6	10	1	1	8	4
**LB Rif**	**10^{-1}**	0	0	0	0	1	0	2	1	0	0	0	0
**LB mean**		567	552	571	575	416	439	361	359	16	74	165	273
**LB Rif mean**		0	0.5	3	2	6	11	13	10	0.5	0.5	4	2
**Ratio [LB Rif/LB]**		0	9.1 $*10^{-10}$	5.3 $*10^{-9}$	3.5 $*10^{-9}$	1.4 $*10^{-8}$	2.5 $*10^{-8}$	3.6 $*10^{-8}$	2.8 $*10^{-8}$	3.1 $*10^{-8}$	6.8 $*10^{-9}$	2.4 $*10^{-8}$	7.3 $*10^{-9}$
**Mean ratio**		**2.4 $* 10^{-9}$**				**2.6 $* 10^{-8}$**				**1.7 $* 10^{-8}$**			

Table S2 | Oligonucleotides used in this study.

ID	Sequence (5´-> 3´)	Purpose
oDS_892	GTCCCTATCTATTAATCATCAGAA	Forward oligo for construction of gRNA. Integration of 3’ homology flank. Targeting intergenic region between PN96_16275 and PN96_16280.
oDS_893	AAACTTCTGATGATTAATAGATAG	Reverse oligo for construction of gRNA. Integration of 3’ homology flank. Targeting intergenic region between PN96_16275 and PN96_16280.
oDS_1420	GTCCGATCGGCAAGCAAAAACAAC	Forward oligo for construction of gRNA. Integration of 5’ homology flank. Targeting intergenic region between PN96_16260 and PN96_16265.
oDS_1421	AAACGTTGTTTTTGCTTGCCGATC	Reverse oligo for construction of gRNA. Integration of 5’ homology flank. Targeting intergenic region between PN96_16260 and PN96_16265.
oDS_904	GTTACAGCGTCACACATTTACATTG	Construction of tDNA for integration of 3’ homology flank. Forward primer of upstream fragment.
oDS_905	TTTTGAGTCAACTTTAAATCGCCATTTCATCAAGAGC	Construction of tDNA for integration of 3’ homology flank. Reverse primer of upstream fragment.
oDS_906	GCGTTATGCTTTGAATAGACTTCGCGAGTTTCTG	Construction of tDNA for integration of 3’ homology flank. Forward primer of downstream fragment.
oDS_907	GAAAGTTGACAAAAACATTAACCATTGAAG	Construction of tDNA for integration of 3’ homology flank. Reverse primer of downstream fragment.
oDS_924	ATGGCGATTTAAAGTTGACTCAAAAGAACCCAGCATACG	Construction of tDNA for integration of 3’ homology flank. Forward primer of insert fragment.
oDS_925	GCGAAGTCTATTCAAAGCATAACGCTACGCCAAATCTTTACTC	Construction of tDNA for integration of 3’ homology flank. Reverse primer of insert fragment.
oDS_896	TGACTTCATCACCAACATTGACAG	Construction of tDNA for integration of 5’ homology flank. Forward primer of upstream fragment.
oDS_1427	CTTTTAACCCTTTGTTGTTTTTGCTTGCCGATCAG	Construction of tDNA for integration of 5’ homology flank. Reverse primer of upstream fragment.
oDS_1430	TATAGTAGACTGTGGCATAAAAAAACCGCCCGAAG	Construction of tDNA for integration of 5’ homology flank. Forward primer of downstream fragment.
oDS_899	ACCATGGATTTTATCGCCTCCTTG	Construction of tDNA for integration of 3’ homology flank. Reverse primer of downstream fragment.
oDS_1428	AGCAAAAACAACAAAGGGTTAAAAGATTAACAGTGCTG	Construction of tDNA for integration of 3’ homology flank. Forward primer of upstream fragment.
oDS_1429	TTTTTTTATGCCACAGTCTACTATAACCCGTATGG	Construction of tDNA for integration of 3’ homology flank. Reverse primer of insert fragment.
oDS_920	CTTTCGCCCTCTAATAGAGC	Primer for cPCR. Confirming integration of 3’ homology flank. Binds in 3’ homology flank.

ID	Sequence (5´-> 3´)	Purpose
oDS_921	GGTATCCAGCAAAGTCTGTC	Primer for cPCR. Confirming integration of 3' homology flank. Binds in chr2 sequence downstream of integration site.
oDS_914	GGGATCCCTTTAGTAGCAGG	Primer for cPCR. Confirming integration of 5' homology flank. Binds in chr2 sequence upstream of integration site.
oDS_915	CCAGAGCGAAGCATGAATCC	Primer for cPCR. Confirming integration of 5' homology flank. Binds in 5' homology flank.
oDS_916	GGATTCATGCTTCGCTCTGG	Primer for cPCR. Confirming integration of 5' homology flank.
oDS_917	CATGACATTTGTACAGGTTATCC	Primer for cPCR. Confirming integration of 5' homology flank. Binds in *oriII* region on chr2.
oDS_1462	ACCCGGAAAGATGATCAAGG	Forward primer to generate PCR fragment for Sanger sequencing to confirm chromosome fusion.
oDS_1464	AATCAGGTGAATTTATTAATGTAAAGGAC	Forward primer to generate PCR fragment for Sanger sequencing to confirm chromosome fusion.
oDS_1463	CCAAGCAAAGTTATTAAGCTCAGC	Sequencing primer to confirm chromosome fusion.
oDS_1477	CATTATGTTTAATGAAAGCCTCAAACC	Sequencing primer to confirm chromosome fusion.
oDS_1460	GGGTGCCTTGCGATATAGC	Forward primer to generate PCR fragment for Sanger sequencing to confirm chromosome fusion.
oDS_1461	ATTCACCGACACAAAACCAACG	Reverse primer to generate PCR fragment for Sanger sequencing to confirm chromosome fusion.
oDS_1455	GAACGAAGTGATAAGTTCGTTTTGC	Sequencing primer to confirm chromosome fusion.
oDS_1459	AAGTAGGGTACTTGGAACTTTTCC	Sequencing primer to confirm chromosome fusion.

Table S3 | Sequencing data deposited under BioProject PRJNA948340.

IDs	Type of data	Platform	Strain	Additional information
SAMN35035422	Raw reads	Illumina (150 PE)	*V. natriegens* Δ*dns*	exponential growing sample – raw data presented in the preprint version of [5]
SAMN35035423	Raw reads	Illumina (150 PE)	*V. natriegens* Δ*dns*	stationary phase sample – raw data presented in the preprint version of [5]
SAMN35035424	Raw reads	Illumina (150 PE)	*V. natriegens* synSC1.0	exponential growing sample – raw data presented in the preprint version of [5]
SAMN35035425	Raw reads	Illumina (150 PE)	*V. natriegens* synSC1.0	stationary phase sample – raw data presented in the preprint version of [5]
SAMN35035426	Raw reads	Nanopore (MinION)	*V. natriegens* Δ*dns*	stationary phase sample
SAMN35035427	Raw reads	Nanopore (MinION)	*V. natriegens* synSC1.0	stationary phase sample
SAMN35394728	Reference	Illumina (150 PE) and Nanopore (MinION)	*V. natriegens* synSC1.0	sequencing validated reference
SAMN35394729	Reference	Illumina (150 PE) and Nanopore (MinION)	*V. natriegens* Δ*dns*	sequencing validated reference
SAMN39954569	Raw reads	Illumina (150 PE)	*V. natriegens* Δ*dns*	exponential growing sample – replicate 1
SAMN39954570	Raw reads	Illumina (150 PE)	*V. natriegens* Δ*dns*	exponential growing sample – replicate 2
SAMN39954571	Raw reads	Illumina (150 PE)	*V. natriegens* Δ*dns*	exponential growing sample – replicate 3
SAMN39954572	Raw reads	Illumina (150 PE)	*V. natriegens* Δ*dns*	stationary phase sample – replicate 1
SAMN39954573	Raw reads	Illumina (150 PE)	*V. natriegens* Δ*dns*	stationary phase sample – replicate 2
SAMN39954574	Raw reads	Illumina (150 PE)	*V. natriegens* Δ*dns*	stationary phase sample – replicate 3
SAMN39954575	Raw reads	Illumina (150 PE)	*V. natriegens* synSC1.0	exponential growing sample – replicate 1
SAMN39954576	Raw reads	Illumina (150 PE)	*V. natriegens* synSC1.0	exponential growing sample – replicate 2
SAMN39954577	Raw reads	Illumina (150 PE)	*V. natriegens* synSC1.0	exponential growing sample – replicate 3
SAMN39954578	Raw reads	Illumina (150 PE)	*V. natriegens* synSC1.0	stationary phase sample – replicate 1
SAMN39954579	Raw reads	Illumina (150 PE)	*V. natriegens* synSC1.0	stationary phase sample – replicate 2
SAMN39954580	Raw reads	Illumina (150 PE)	*V. natriegens* synSC1.0	stationary phase sample – replicate 3

Supplementary References

1 Val, M. E., Skovgaard, O., Ducos-Galand, M., Bland, M. J. & Mazel, D. Genome engineering in *Vibrio cholerae*: A feasible approach to address biological issues. *PLoS Genet* **8**, e1002472, doi:10.1371/journal.pgen.1002472 (2012).

2 Bruhn, M. *et al.* Functionality of two origins of replication in *Vibrio cholerae* strains with a single chromosome. *Front Microbiol* **9**, 2932, doi:10.3389/fmicb.2018.02932 (2018).

3 Galli, E. *et al.* Cell division licensing in the multi-chromosomal *Vibrio cholerae* bacterium. *Nat Microbiol* **1**, 16094, doi:10.1038/nmicrobiol.2016.94 (2016).

4 Galli, E. *et al.* Replication termination without a replication fork trap. *Sci Rep* **9**, 8315, doi:10.1038/s41598-019-43795-2 (2019).

5 Ramming, L., Stukenberg, D., Sánchez Olmos, M. d. C., Becker, A. & Schindler, D. DNA replication is not a limiting factor for rapid growth of *Vibrio natriegens*. *bioRxiv*, doi:10.1101/2023.05.26.541695 (2023).

6 Müller, C. A. *et al.* The dynamics of genome replication using deep sequencing. *Nucleic Acids Res* **42**, e3, doi:10.1093/nar/gkt878 (2014).

7 Lee, H. H. *et al.* Functional genomics of the rapidly replicating bacterium *Vibrio natriegens* by CRISPRi. *Nat Microbiol* **4**, 1105-1113, doi:10.1038/s41564-019-0423-8 (2019).
