# Supplementary material for: Rationally designed chromosome fusion does not prevent rapid growth of Vibrio natriegens
Source: Commun Biol. 2024 May 2;7:519. doi: 10.1038/s42003-024-06234-1 (PMC11066055; doi:10.1038/s42003-024-06234-1)
Supplement: Supplementary file 2 — Description of Additional Supplementary Files [file 42003_2024_6234_MOESM2_ESM.docx]

**Description of Additional Supplementary Files**

**File name:** Supplementary Data S1

**Description:** Replication pattern analysis values.

**File name:** Supplementary Data S2

**Description:** GenBank files of plasmids and tDNAs.

**File name:** Supplementary Data S3

**Description:** Shotgun proteome quantification data.

**File name:** Supplementary Data S4

**Description:** Numerical raw data of growth and cell size measurements.
